# Effects of nerve growth factor (NGF) on blood vessels area and expression of the angiogenic factors VEGF and TGFbeta1 in the rat ovary

**DOI:** 10.1186/1477-7827-4-57

**Published:** 2006-11-10

**Authors:** Marcela Julio-Pieper, Hernán E Lara, Javier A Bravo, Carmen Romero

**Affiliations:** 1 Laboratory of Endocrinology and Reproductive Biology, Hospital Clínico Universidad de Chile, Santiago, Chile; 2 Laboratory of Neurobiochemistry, Faculty of Chemical and Pharmaceutical Sciences, Universidad de Chile, Santiago, Chile

## Abstract

**Background:**

Angiogenesis is a crucial process in follicular development and luteogenesis. The nerve growth factor (NGF) promotes angiogenesis in various tissues. An impaired production of this neurotrophin has been associated with delayed wound healing. A variety of ovarian functions are regulated by NGF, but its effects on ovarian angiogenesis remain unknown. The aim of this study was to elucidate if NGF modulates 1) the amount of follicular blood vessels and 2) ovarian expression of two angiogenic factors: vascular endothelial growth factor (VEGF) and transforming growth factor beta 1 (TGFbeta1), in the rat ovary.

**Results:**

In cultured neonatal rat ovaries, NGF increased VEGF mRNA and protein levels, whereas TGFbeta1 expression did not change. Sectioning of the superior ovarian nerve, which increases ovarian NGF protein content, augmented VEGF immunoreactivity and the area of capillary vessels in ovaries of prepubertal rats compared to control ovaries.

**Conclusion:**

Results indicate that NGF may be important in the maintenance of the follicular and luteal vasculature in adult rodents, either indirectly, by increasing the expression of VEGF in the ovary, or directly via promoting the proliferation of vascular cells. This data suggests that a disruption on NGF regulation could be a component in ovarian disorders related with impaired angiogenesis.

## Background

Angiogenesis is an essential process in follicular development and luteogenesis [1,2]. Once the proliferation of new blood vessels is complete, a rapid capillary regression takes place in the non-fertile cycle which suggests a delicate coordination between angiogenesis inducers and inhibitors [3]. The intervention of ovarian vascularization has an adverse effect on the growth of the dominant follicle, the ovulation and the functioning of the corpus luteum and its ability to secrete progesterone [1,4].

The regulation of cyclic angiogenesis in the ovary is commanded by a variety of growth factors; vascular endothelial growth factor (VEGF) playing a major role by stimulating vessel hyperpermeability, endothelial cell proliferation and migration [5]. Several splicing isoforms are generated from VEGF gene, and the proteins reach endothelial cells either by difussion of the shorter isoforms (VEGF 121, VEGF 165) or following proteolitic cleavage of longer ones (VEGF 189, VEGF 206) [3]. The mRNAs for the isoforms of 121 and 165 aminoacids are dominant in normal human ovaries [5]. In rodents, VEGF isoforms are one aminoacid shorter than in human, and have a similar distribution and function [6,7]. Another molecule that modulates angiogenesis is the transforming growth factor beta 1 (TGFβ1), being essential for matrix remodeling, vessel stabilization and pericyte differentiation. TGFβ1 deficient mice have impaired vessel wall integrity [8,9]. In the human ovary, follicular cells express TGFβ1, which has proliferative and differentiation effects on granulosa cells [10,11]. It also increases the size of follicles from adult mice [12]. In the hamster ovary, the mRNAs for TGFβ receptors are cyclically regulated by gonadotropins and ovarian steroids, increasing their levels as the follicle develops during the estral cycle [13].

Another important growth factor involved in ovarian physiology is the nerve growth factor (NGF). Mammalian ovary expresses the neurotrophin and both the high- and the low-affinity receptor for NGF (trkA and p75, respectively) [14-16]. In neonatal rat ovaries, the expression of follicular stimulating hormone (FSH) receptors is induced by NGF [17]. Immunoneutralization of NGF during early postnatal life of the rat results in undersized antral follicles, delayed puberty and disrupted estrous cyclicity [18]. TrkA and NGF are transiently expressed in preovulatory follicles in the first preovulatory surge of gonadotropins at puberty in the rat. The use of a neutralizing antibody to NGF or pharmacological blockade of trkA tyrosine kinase activity targeted to one ovary resulted in the ipsilateral inhibition of ovulation [19].

Although NGF promotes angiogenesis and/or induces the expression of angiogenic molecules in several tissues, such as skin, muscle, cornea, arteries [20-23] and the immunoneutralization of this neurotrophin delays the reparative neovascularization of lesions in the mice [21], the effects of NGF on ovarian angiogenesis remain unexplored. This study investigates the role of NGF in regulating the expression of the angiogenic factors VEGF and TGFβ1, and the area of blood vessels in the rat ovary. Gonadotropins can upregulate different angiogenesis-related parameters in the ovary [24-26]; hence, to avoid this influence, we selected early stages of rat ovarian life as a model, when the tissue has not become gonadotropin-dependent [27].

## Methods

### Animals

Sprague Dawley rats obtained from the Faculty of Chemical and Pharmaceutical Sciences, Universidad de Chile were housed under controlled conditions of temperature (21°C) and light (12 h of light, 12 h of darkness; lights on from 0800–2000 h). Food and drinking water were provided *ad libitum*. All the protocols for animal care and use included in this study were approved by the institutional review board of the Faculty of Chemical and Pharmaceutical Sciences, Universidad de Chile.

### Organ culture

Ovaries from 2 day old rats were dissected under aseptic conditions, placed on sterile lens paper, and cultured on plastic supports at the interface of air/culture medium [28], under an atmosphere of 5% CO2. For histochemical studies, one ovary per well was utilized, and for mRNA studies, three glands per well were cultured. Considering this, for an n = 5, 20 pups were utilized for each incubation point. Ovaries were cultured in 24-well plates (Becton Dickinson, USA); each well contained 1 ml DMEM: F-12 (50% vol/vol, Sigma Chemicals, St Louis, MO, USA) medium supplemented with sodium bicarbonate (600 mg/l, Sigma Chemicals, St Louis, MO, USA), penicillin (50 mg/l, Laboratorio Chile, Santiago, Chile), gentamycin (80 mg/l, Andrómaco, Santiago, Chile), streptomycin (50 mg/l, Laboratorio Chile, Santiago, Chile) and ketoconazol (5 mg/l, Laboratorio Chile, Santiago, Chile). The ovaries were stimulated with NGF 100 ng/ml (Sigma Chemicals, St Louis, MO, USA). The optimal dose of NGF was established by previous studies from our group [17]. The contralateral ovaries cultured with medium alone were used as controls. The times of culture were 2, 4, 8 and 24 h. In the case of mRNA studies, the ovaries were collected and stored at -80°C until RNA extraction. The ovaries collected for immunohistochemistry were fixed in Bouin's fixative.

### Ovary denervation

We used 16 prepuberal rats of 23–24 day old (body weight 60 g aprox.). They were anaesthetized with isoflurane 1% v/v, 2.5 l/min (Baxter Healthcare Co, Guayama, Puerto Rico). The dissection of the superior ovarian nerve (SON) was performed in both ovaries under aseptic conditions through a single dorsal midline incision. This procedure has shown to induce an increase of NGF within the gland [29]. Because denervation induces hypertrophy of the contralateral ovary [30], controls consisted in different animals submitted to a simulated operation. The ovaries were collected three days later (from a total of 6 rats; 3 controls and 3 denervated) or six days later (from a total of 10 rats; 5 controls and 5 denervated), and stored at -80°C for mRNA studies, or fixed in Bouin's fixative for histochemical procedures. The time for ovary harvesting after denervation was established by previous studies from our group [29].

### Immunohistochemistry

Immunohistochemical detection of VEGF and TGFβ1 in denervated ovaries and cultured tissues was performed as follows: the ovaries were fixed by immersion in Bouin's fixative, embedded in paraffin, serially sectioned at 4 μm and processed for immunohistochemistry using polyclonal antibodies from Santa Cruz Biotechnology (anti-VEGF [C-1], sc-7269 and anti-TGFβ1 [V] sc-146, both in a 1:50 dilution). Tissue sections were incubated overnight at 4°C with the antibody and the immunoreaction was developed the next day using the NBT-BCIP procedure (Sigma Chemicals, St Louis, MO, USA) for VEGF in neonatal rat ovaries, or the DAB procedure (LabVision Co, Freemont CA, USA) for the rest of the detections. Controls consistent of adjacent sections incubated without the primary antibody, or sections incubated with the antibody preabsorbed with the corresponding ligand showed no staining, proving the specificity of the immunoreaction. Whenever a well defined mark was obtained, cell counting was performed by three independent observers and the data were expressed as H-Score. The H-Score is the sum of the proportion of cells showing different degrees of reactivity, and was calculated as follows: 0 times the % of negative cells + 1 time the % of weakly positive cells + 2 times the % of moderately positive cells + 3 times the % of strongly positive cells. The H-Score ranges from 0–300, being the maximum score representative of a 100% of cells with strong positivity [31]. In the case of diffuse mark, staining intensity was evaluated with an automated digitizing system (UN-SCAN-IT gel 4.1, Silk Scientific Co, USA) and the data were expressed as number of pixels.

Immunohistochemical detection of endothelial cell marker PECAM-1 in denervated ovaries was performed using an antibody from Santa Cruz Biotechnology (anti-PECAM-1 [M-20] in a 1:100 dilution). Tissue sections were incubated with the antibody overnight at 4°C and the immunoreaction was developed the next day using the DAB procedure (LabVision Co, Freemont CA, USA). Controls consisted of adjacent sections incubated without the primary antibody. The area of positive staining was evaluated with an automated system (Image J 1.36b, NIH, USA) and the data were expressed as % area of the ovary section.

### RNA extraction and reverse transcription reaction

Total RNA was extracted from rat ovaries using guanidine isothiocyanate and phenol-chloroform extraction (Trizol Reagent, Invitrogen, Foster City CA, USA) following the manufacturer's protocol. Concentration and purity of RNA were measured using a spectrophotometer at 260 and 280 nm. First strand cDNA was synthesized in a 20 ml reaction mixture using 1 μg of total RNA. The reaction tubes contained 0.5 μl random hexamers (500 ng/μl, Invitrogen, Foster City CA, USA), 1 μl dNTPs (10 mM, Invitrogen, Foster City CA, USA), 4 μl 5× reaction buffer (250 mM Tris-HCl pH 8.3, 375 mM KCl, 15 mM MgCl2, Invitrogen, Foster City CA, USA), 2 μl DTT (0.1 M, Invitrogen, Foster City CA, USA), 1 μl ribonuclease inhibitor (10 U/μl, Invitrogen, Foster City CA, USA) and 1 μl M-MLV reverse transcriptase (200 U/μl, Invitrogen, Foster City CA, USA). Reactions were incubated at 37°C for 60 minutes and inactivated by freezing.

### Polymerase chain reaction

The specific primer sequences for the examined genes and the predicted PCR product sizes are shown in Table 1. The cDNA was amplified in a 25 μl reaction mixture using 1 μl of single-strand cDNA. PCR conditions were as follows: 2.5 μl 10 × reaction buffer (200 mM Tris-HCl pH 8.4, 500 mM KCl, Biotools, Madrid, Spain), 1 μl MgCl2 (50 mM), 0.5 μl dNTPs (10 mM each), 1.25 μl each primer (10 μM, Polyscience, Santiago, Chile), 0.15 μl DNA polymerase (5 U/μl, Biotools, Madrid, Spain). Reaction mixtures were incubated in a thermal cycler (Eppendorf, Foster City CA, USA) for 2 minutes at 94°C. Then 23, 27, or 28 cycles were performed for cyclophilin, VEGF and TGFβ1 respectively including: denaturation at 94°C for 45 seconds, annealing at 62°C for 1 minute and extension at 72°C for 1 minute. The optimal number of cycles was experimentally determined by analyzing the exponential phase of amplification reaction (data not shown). The PCR products were separated on Tris-borate-EDTA 2% agarose gels containing 200 ng/ml ethidium bromide (Invitrogen, Foster City CA, USA). DNA size markers were run in parallel to validate the predicted sizes of the amplified bands (100-bp DNA ladder, Biotools, Madrid, Spain). The gels were visualized under UV light, photographed using a capturing program (Ultra Violet Products Doc-It), and analyzed with an automated digitizing system (UN-SCAN-IT gel 4.1, Silk Scientific Corporation, Orem UT, USA). Differences between the experimental versus control conditions, were obtained by the ratio of the intensity between specific gene and constitutive gene of each sample, determined by densitometry.

**Table 1:** Primer sequences used for PCR of cDNAs

Gene	Forward and reverse primers (5'– 3')	PCR product size (bp)	Reference
VEGF	F: GCCAGCACATAGGAGAGATGAG R: ACCGCCTTGGCTTGTCAC	234, 102	Adapted from [45]
TGFβ1	F: AAGTGGATCCACGAGCCCAA R: GCTGCACTTGCAGGAGCGCA	246	[46]
Cyclophilin	F: CTTTGCAGACGCCGCTGTCTCTTTTCGCCG R: GCATTTGCCATGGACAAGATGCCAGGA	350	[47]

### Statistical analysis

The differences in mRNA levels, immunoreactivity and area of positive staining were analyzed using Mann Whitney test.

## Results

### NGF increases VEGF mRNA and protein levels in neonatal rat ovaries

**Figure 1:**
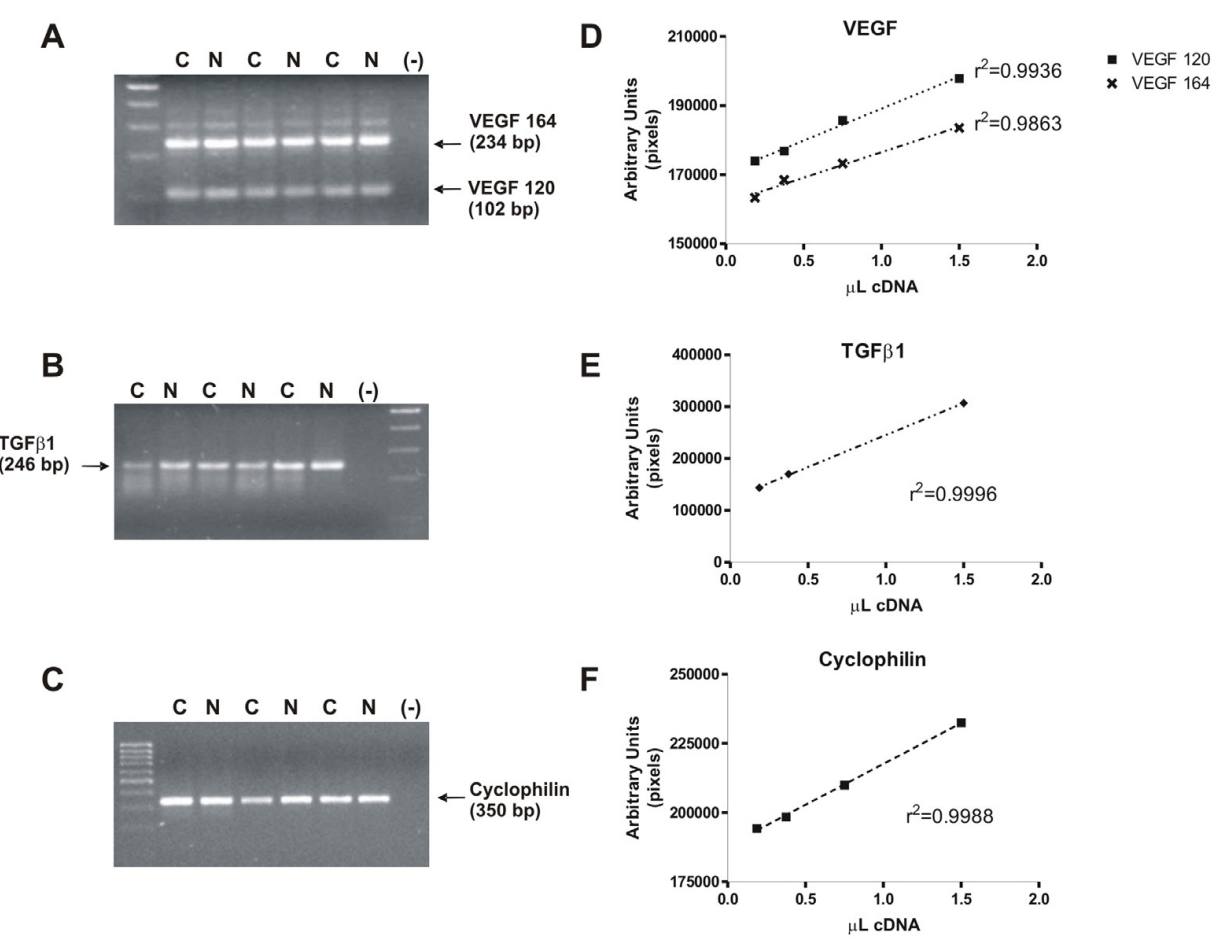
**PCR products for the cDNAs evaluated in this study**. Pannels A, B and C show photographs of agarose electrophoresis for representative PCR products of the different target sequences studied: Pannel A shows PCR products for VEGF (102 bp for VEGF 120 and 234 bp for VEGF 164) of control samples (C) and NGF treated ovaries (N); additional bands corresponding to other VEGF isoforms were also visible for some samples and thus could not be considered in this study. Pannel B shows PCR product for TGFβ1 (246 bp) of control (C) and NGF treatment (N); Pannel C shows PCR products for the constitutive gene Cyclophilin (350 bp) of control (C) and NGF treatment (N). Negative controls in each photograph correspond to the lane indicated as (-). Pannels D, E and F represent graphs of PCR reactions starting with different quantities of cDNA, indicating the linear range in which each densitometry was made in order to observe differences in each experimental condition: D) Linear range for VEGF amplification (r^2 ^= 0.9936 for VEGF 120 and r^2 ^= 0.9863 for VEGF 164); E) Linear range for TGFβ1 amplification (r^2 ^= 0.9996); F) Linear range for Cyclophilin amplification (r^2 ^= 0.9988). Each graph represents PCR products obtained from a pool of cDNA samples.

The photograph of 2% agarose gel electrophoresis of representative PCR products for VEGF120, VEGF164, TGFβ1 and Cyclophilin (as constitutive gene) are shown in figure 1A-C. The exponential phase of amplification for each target sequence was determined by performing PCR under different number of cycles (ranging from 20 to 35 cycles). The PCR products were separated in agarose gels, stained with ethidium bromide, and the intensity of the bands was analyzed with an automated digitalizing program (UN-SCAN-IT gel 4.1, Silk Scientific Corporation, Orem UT, USA). Results were plotted to visualize the exponential region of the curve, and thus the optimal number of cycles was selected for each target sequence (not shown). A dose/response curve was performed for each target sequence by adding increasing amounts of cDNA to the respective PCR mix. The response showed to be linear under working conditions (see figure 1D-F).

**Figure 2:**
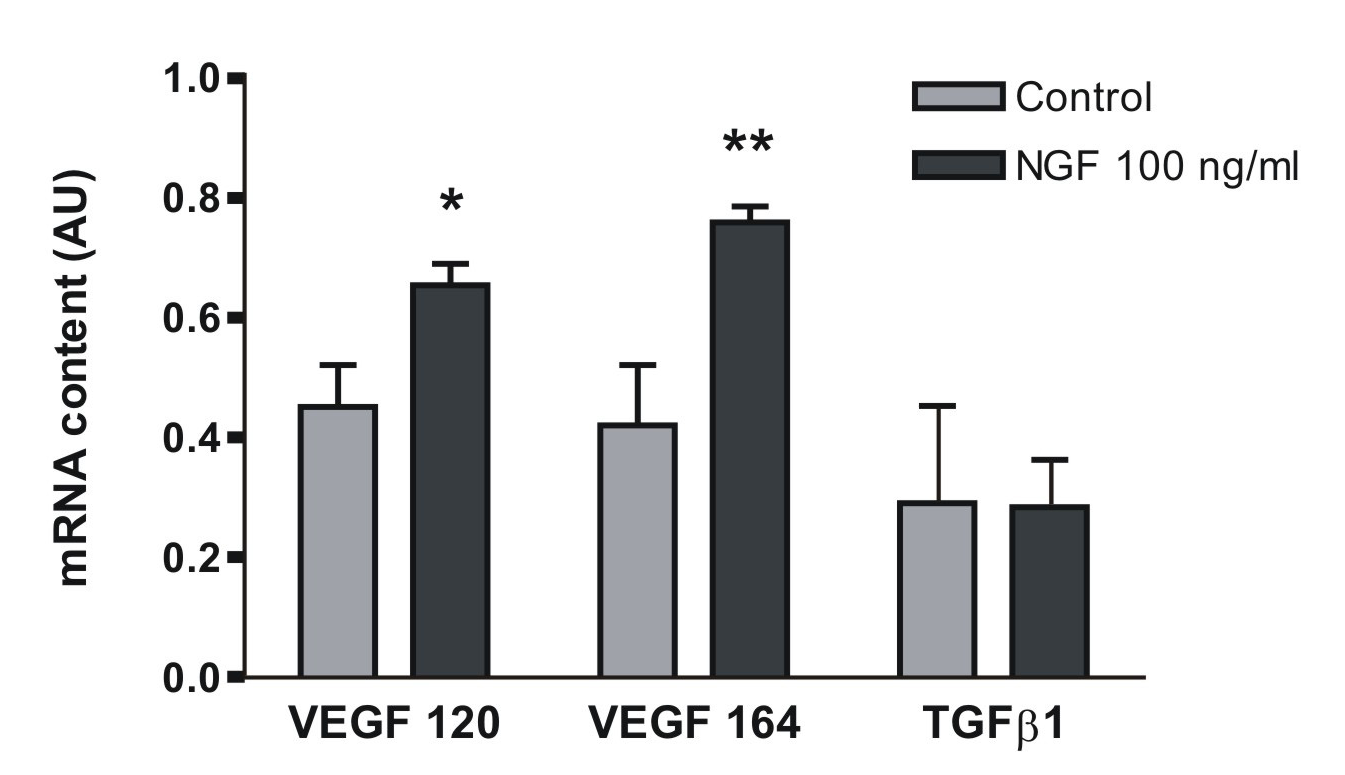
**mRNA content of VEGF isoforms and TGFβ1 in neonatal rat ovaries cultured for 2 hours with NGF**. The results were evaluated by densitometric analysis of PCR products bands and expressed as arbitrary units (AU, pixels from specific gene/pixels from constitutive gene) mean ± SEM from n = 5. * p < 0.05 and ** p < 0.01 vs control.

Neonatal rat ovaries were cultured with NGF (100 ng/ml) for 2, 4, 8 and 24 h. The mRNA levels for VEGF 120 and VEGF 164 – the most relevant VEGF isoforms in ovarian physiology – exhibited a significant increase in NGF-stimulated tissues compared to controls, in the two-hour culture, as shown in figure 2. No change on TGFβ1 mRNA content was found at any assayed stimulation time.

**Figure 3:**
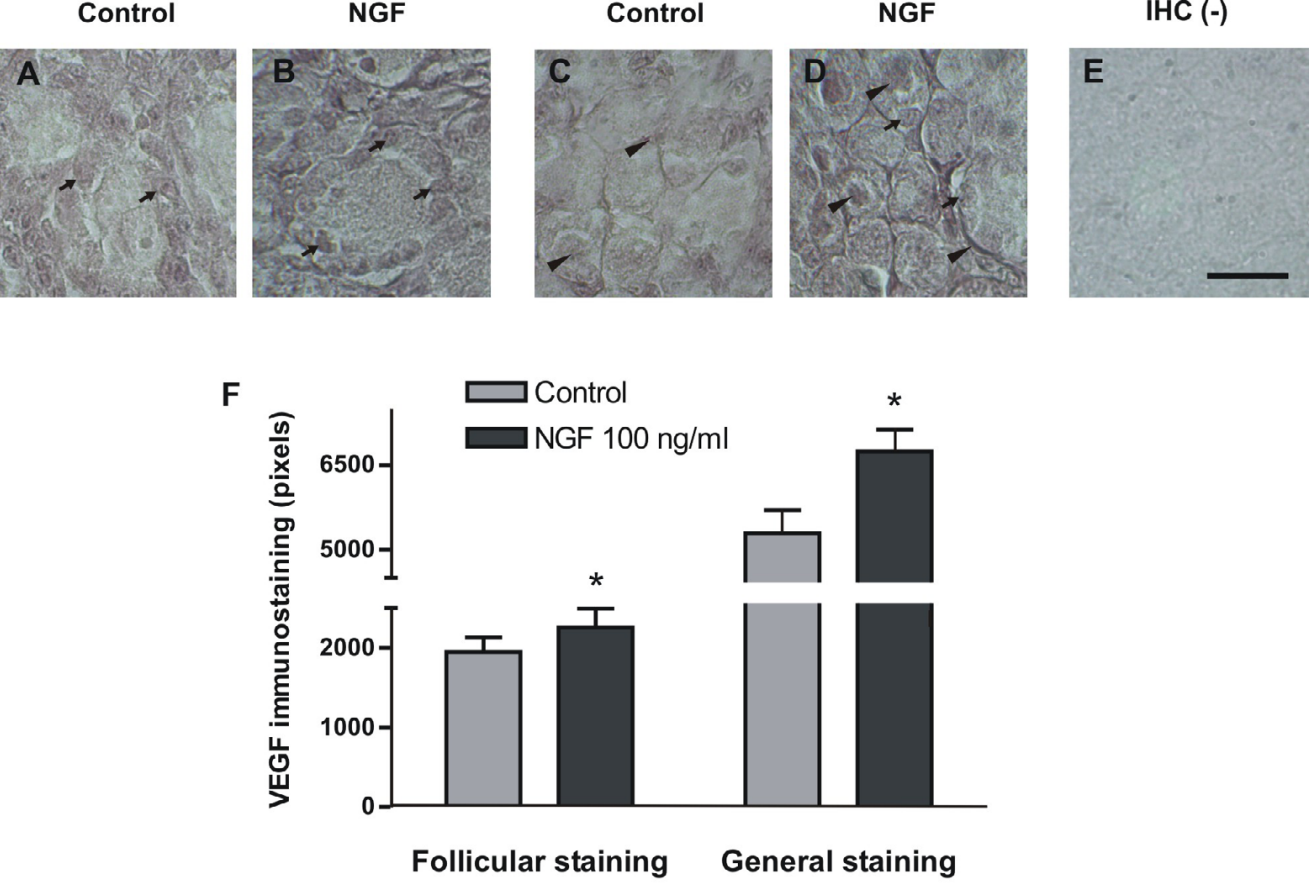
**VEGF immunostaining in neonatal rat ovaries cultured for 24 hours with NGF**. Arrows depict the predominant location of VEGF in granulosa cells of the ovarian follicles (A-B). Arrowheads indicate the presence of VEGF in other cell types of the tissue (C-D), such as oocytes. A and C show the staining of representative control ovaries, and B and D the respective NGF-treated tissues. A negative immunohistochemistry is shown in E. Mean ± SEM for densitometry from n = 5, are shown in F. * p < 0.05 vs control. Original magnification 100×. Bar = 50 μm.

**Figure 4:**
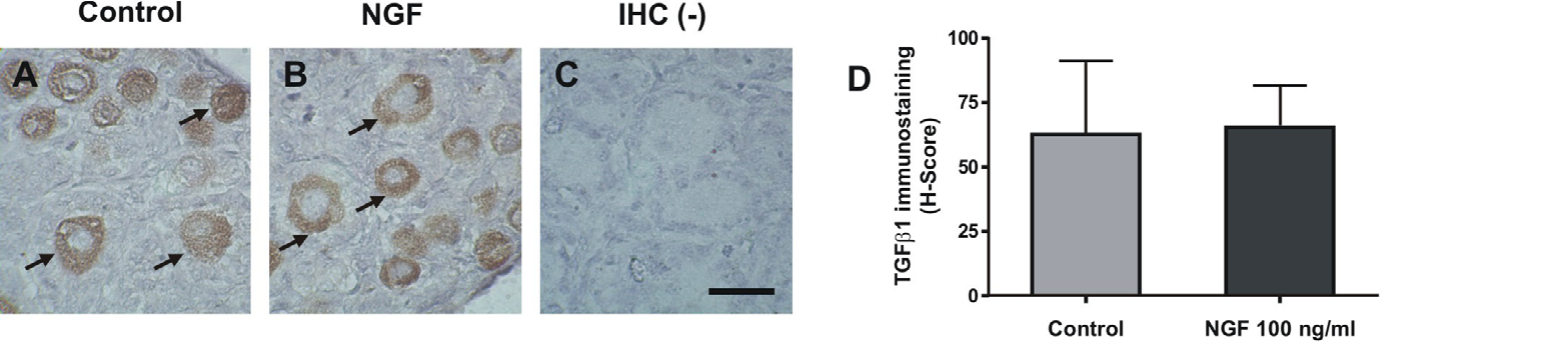
**TGFβ1 immunostaining in neonatal rat ovaries cultured for 24 hours with NGF**. Arrows depict the predominant location of TGFβ1 at the oocytes. A and B show the staining of control and NGF-treated ovaries, respectively. A negative immunohistochemistry is shown in C. Mean ± SEM for H-Score from n = 5 are shown in D. Original magnification 100×. Bar = 50 μm.

Regarding localization of VEGF protein in neonatal rat ovaries, although two controls for specificity were used, immunoreactivity was present more specifically in granulosa cell layer of primary follicles (figures 3A and 3B) and also in other types of ovarian cells (figures 3C and 3D). Densitometric analysis of the immune signal revealed that after 24 hour incubation, NGF induced an increase in VEGF staining in neonatal rat ovaries, which was significant both for the overall staining of the ovarian tissue and for staining of primary follicles (figure 3F). No significant differences in VEGF staining were found in shorter incubations (2, 4 or 8 h). As to TGFβ1, a specific mark was detected in oocytes (figure 4A and 4B). The same as for mRNA, H-Score of TGFβ1 immunoreactivity was not modified by NGF (figure 4D).

### Denervation of the ovary increases VEGF immunoreactivity in prepubertal rats

**Figure 5:**
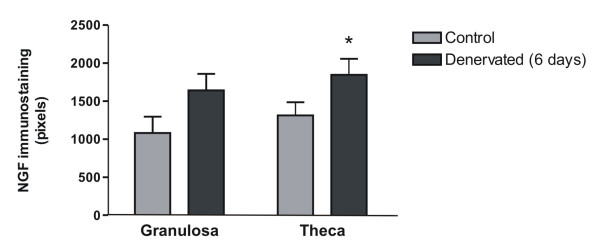
**NGF immunostaining in rats with a dissection of the superior ovarian nerve (SON)**. Positive reaction was evaluated by densitometry and results were expressed as mean ± SEM from n = 5. * p < 0.05 vs control.

**Figure 6:**
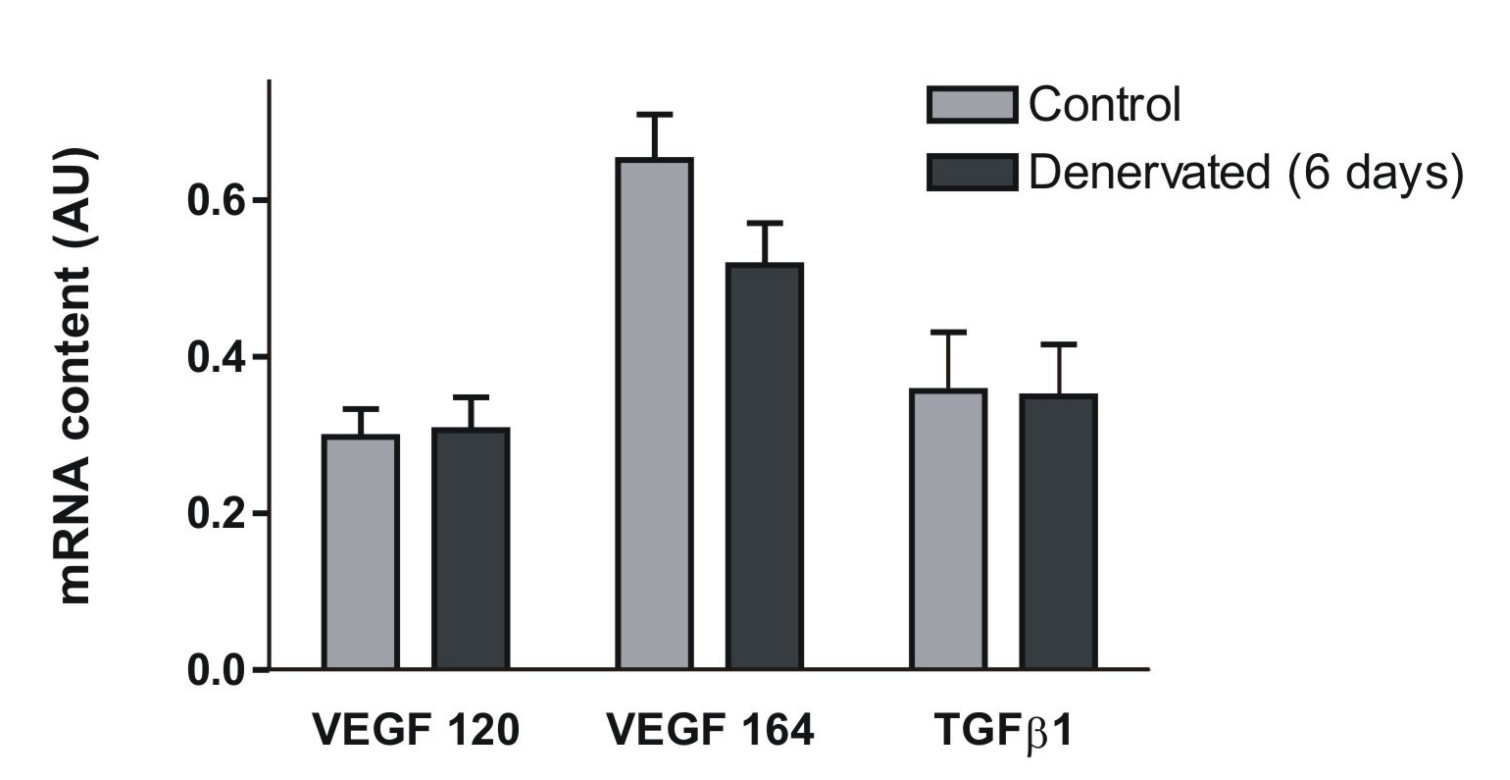
**mRNA content of VEGF isoforms and TGFβ1 in rats with increased ovarian NGF induced by SON dissection**. The results were evaluated by densitometric analysis of PCR products bands and expressed as arbitrary units (AU, pixels from specific gene/pixels from constitutive gene) mean ± SEM from n = 5.

**Figure 7:**
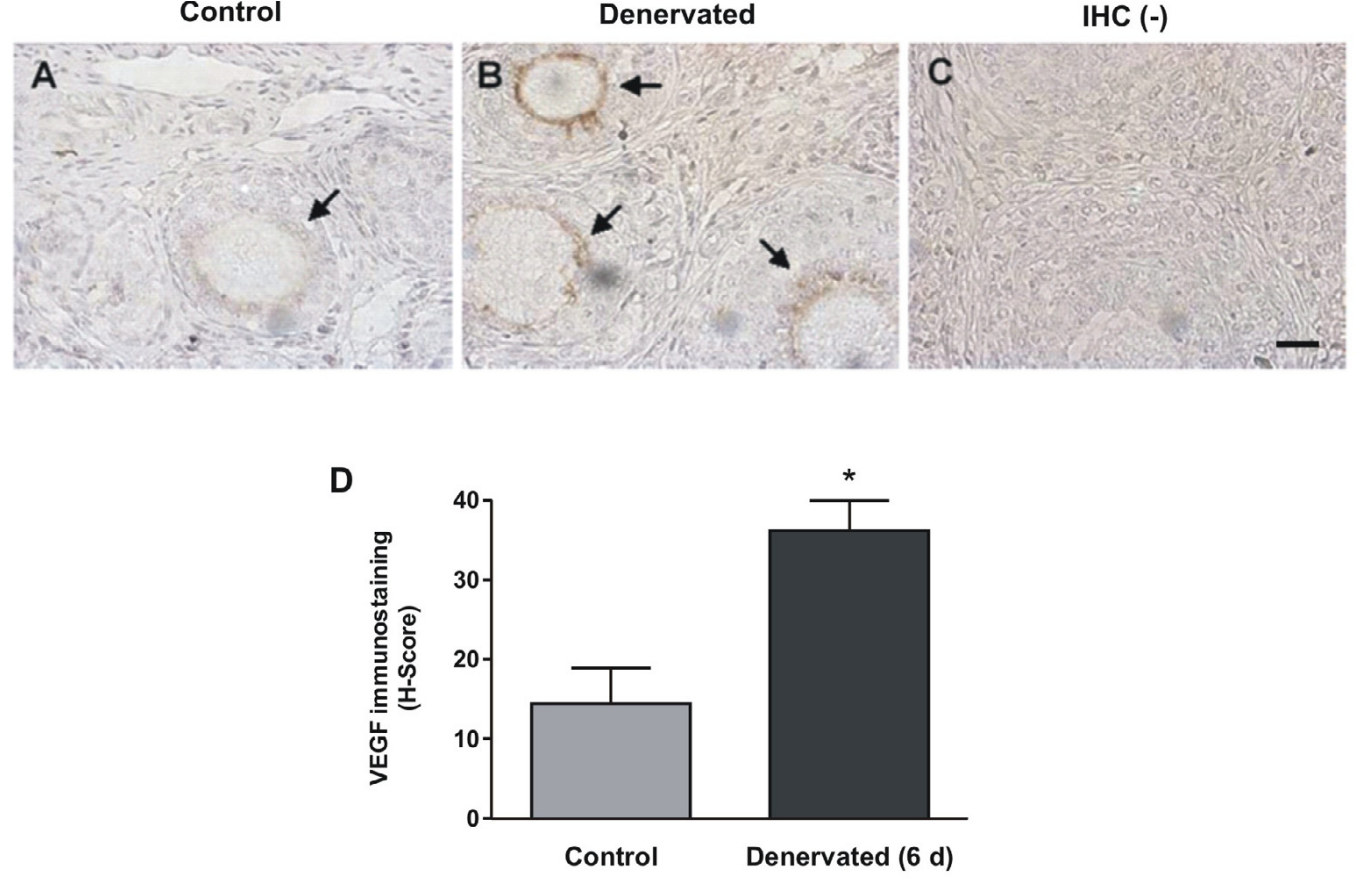
**VEGF immunostaining in rats with increased ovarian NGF as a consequence of SON dissection**. The predominant location of VEGF was the inner layer of the granulosa compartment, as depicted by arrows. A and B show the staining of control and denervated ovaries, respectively. Negative control is shown in C. Mean ± SEM for H-Score from n = 3, are shown in D. * p < 0.05 vs control. Original magnification 40×. Bar = 150 μm.

It was important to determine whether this neurotrophin would increase VEGF in an *in vivo *condition previously informed to provoke an increase in NGF in the rat ovary. We performed a superior ovarian nerve (SON) sectioning in prepubertal rats and confirmed that NGF protein levels, evaluated by densitometry of immunohistochemistry-processed tissues, were increased in theca cell layers of ovaries obtained from denervated animals when compared to control ones (figure 5). Nevertheless, after 6 days of denervation, no change was found in mRNA level of VEGF and TGFβ1 (figure 6). Although there were no differences in mRNA content, H-Score evaluation indicated that the immunoreactivity for VEGF was significantly increased in the inner granulosa cell layer of denervated ovaries as compared with control animals (Figure 7A,B and 7D). TGFβ1 immunoreactivity did not change after denervation (results not shown). To evaluate if there was an earlier change in VEGF and TGFβ1 mRNA levels or in TGFβ1 protein, the same studies were performed on day 3 of SON sectioning. We found no significant difference for any of the markers studied (results not shown).

### Denervation of the ovary increases blood vessels area in prepubertal rats

**Figure 8:**
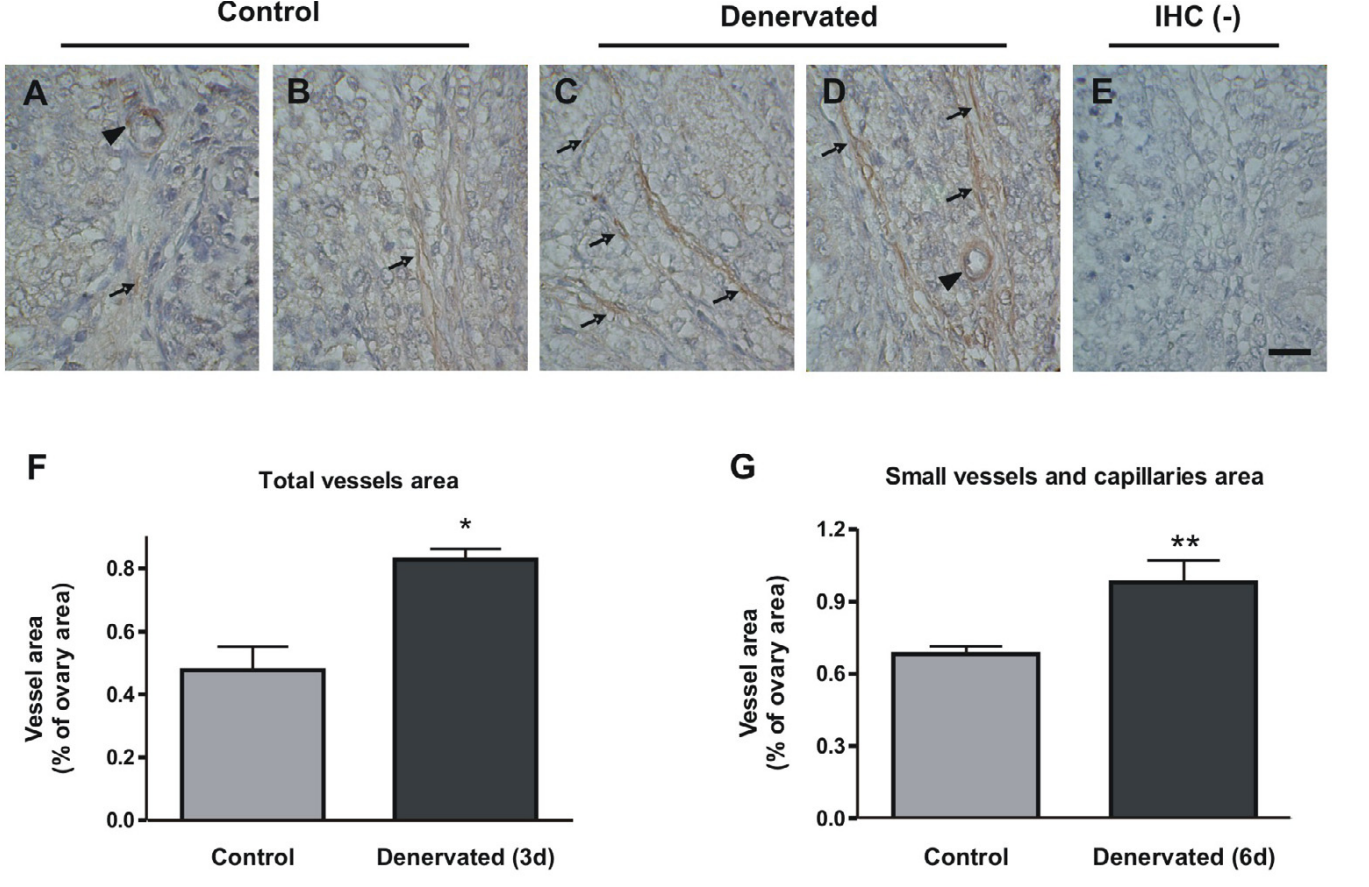
**PECAM-1 immunostaining in rats with increased ovarian NGF as a consequence of SON dissection**. Arrows depict the predominant location of vessels in the area surrounding the ovarian follicles. Small vessels are depicted by arrowheads. A-B show the immunostaining found in control rat ovaries, and C-D show the ovaries of 6-day denervated rats, where a bigger area of capillaries and small vessels was stained. F and G show the quantification of the stained vessels area in rats denervated for 3 (n = 3) and 6 days (n = 5), respectively. The area of vessels positively stained was evaluated with the Image-J program and results were expressed as mean ± SEM. * p < 0.05 and ** p < 0.01 vs control. Original magnification 100×. Bar = 100 μm.

We wanted to evaluate if denervated rats, which have higher ovarian NGF levels, present an increase in ovarian blood vessels. Immunohistochemical studies revealed that the amount of ovarian small vessels and capillaries, evaluated as % area of PECAM-1 positive staining was increased in rats denervated for 6 days, when compared to control animals (figure 8A-D and 8G). A similar result was found in denervated animals as early as three days after SON sectioning (figure 8F).

## Discussion

The present study shows that NGF increased the expression of the angiogenic factor VEGF and the area of blood vessels detected by the endothelial cell marker PECAM-1, making a connection between two essential processes in ovarian physiology, such as angiogenesis and ovulation.

There is an increase of both NGF and its receptor trkA a few hours before ovulation in the rat ovary; this seems to be a critical step in the ovulatory cascade, since the use of a neutralizing antibody to NGF or a blocker of trkA activity inhibits the ovulation [19]. In this periovulatory time and specially following ovulatory rupture, there is period of intensive follicular/luteal vascularization, in which angiogenic growth factors such as VEGF, essential for endothelial cell proliferation, become highly expressed [1,7].

Finding VEGF expression in the neonatal ovary suggests that VEGF could also have actions different from neovascularization in early stages of ovarian development (e.g. proliferation of somatic cells or increased vessel permeability to allow extravasation of nutrients and hormones). NGF induced an early VEGF mRNA increase in neonatal rat ovaries. The explanation of this rapid response could lie on the presence of trkA in the rat ovary shortly after birth [32]. Indeed, NGF has been found necessary not only during the ovulatory process: ovaries from NGF-null mutant mice have a reduced population of primary and secondary follicles, a higher number of oocytes that are not incorporated into follicles, and a reduction in cell proliferation. [33]. Immunoneutralization of NGF during early postnatal life of the rat impairs the growth of antral follicles and delays puberty [18]. In this respect, the NGF/trkA complex may act as a regulator of ovarian VEGF expression in the first days of postnatal life of the rat.

This study shows that NGF can increase two mayor angiogenesis-related parameters within the ovary: 1) the expression of VEGF, an ovarian pro-angiogenic molecule, and 2) the amount of ovarian blood vessels. It remains to be elucidated if the increase in blood vessels is a consequence of NGF binding to trkA receptor in endothelial cells, or is mediated by the increase of VEGF, but it is reported that NGF is able to activate endothelial cell proliferation independent of VEGF [34]. VEGF 120 and VEGF 164 mRNAs did not change after three or six days of SON denervation, a result that could be explained by the early response of neonatal ovaries exposed to NGF.

NGF did not modify TGFβ1 expression, either in neonatal or in prepubertal rat ovaries. This, in addition to previously informed data in the hamster ovary that the expression of TGFβ receptors mRNA changed cyclically [13], suggest that TGFβ1 action on ovarian cycle might be controlled at TGFβ receptor expression rather than ligand level. On the other hand, it is known that NGF is able to modulate the expression of TGFβ receptors in grafted adrenal chromaffin cells, by reducing the level of TβRII mRNA [35]. Then, it would be very interesting to study the NGF effect on TGFβ receptors expression in the ovary to better understand this mechanism.

Defects in angiogenesis regulation can be related with a variety of pathologies, like hemangioma, psoriasis and most of neoplasic conditions [36-38]. Women suffering from polycystic ovaries have an increased ovarian blood flow, which is probably associated with the higher serum VEGF levels found in these patients [39]. An elevation of ovarian NGF and p75 is observed in rats with steroid-induced polycystic ovaries, and the intraovarian administration of a neutralizing antiserum to NGF in conjunction with an antisense to p75 normalized estrous cyclicity and ovulatory capacity in a majority of the animals [40]. It cannot be discarded that fertility disorders like polycystic ovary, or others associated with impaired angiogenesis have a genesis in a deregulation of NGF expression or function that results in aberrant production of VEGF. Finally, our group has demonstrated that VEGF is regulated by NGF in epithelial ovarian cancer [41].

Accumulating evidence about the importance of the neurotrophins and their receptors in ovarian physiology has appeared [14-16]. In addition to NGF, also BDNF, NT3 and NT4 have been described in the neonatal ovary [32,42] and some of them have been associated with early follicular development [32]. NGF and trkA have been involved in processes such as steroidogenesis, FSH receptor expression and proliferation of somatic cells in rodent and human ovary [17,43,44]. Our results are the first to relate NGF with ovarian angiogenesis and confirm the angiogenic effects of NGF in the rat ovary, giving new insight for a role of NGF in the mammalian ovarian function.

## Conclusion

PCR and immunohistochemistry studies showed that NGF can increase VEGF expression in cultured neonatal rat ovaries. This was verified in an *in vivo *model: prepubertal rats with a dissection of the superior ovarian nerve – performed to increase the local levels of NGF – had also increased VEGF immunoreactivity within the ovary on the day six after denervation. TGFβ1 expression was not modified by NGF in any of the models under study.

In prepubertal rats NGF is able to increase the area of ovarian vessels, as shown by endothelial cell staining. The % area of PECAM-1 positive staining was increased in rats denervated for 3 and 6 days, when compared to control animals.

In summary, the present study shows that NGF increases the expression of the angiogenic factor VEGF and the area of blood vessels in the rat ovary, two major events of the periovulatory period that are fundamental for ovarian physiology.

## Competing interests

The author(s) declare that they have no competing interests.

## Authors' contributions

MJ-P carried out the experimental design, performed the tissue cultures, immunohistochemistries and PCR studies, statistical analysis, interpretation of data and drafted the manuscript. HEL performed the rat surgeries and contributed to draft the manuscript. JAB helped with the rat surgeries and contributed to the data analysis and to draft the manuscript. CR participated in the design and coordination of the study, contributed to the data analysis and helped to draft the manuscript. All authors read and approved the final manuscript.
